# Impact of a novel spine stabilization technique on intervertebral movement in cervical spine surgery using a humanoid surgical robotic system

**DOI:** 10.1016/j.xnsj.2026.100928

**Published:** 2026-07-15

**Authors:** Alon Friedlander, Fabrice Külling, Sharon A. Ottens, Yossi Bar, Xavier Camps Mullarach, Andreas Raabe

**Affiliations:** a Department of Orthopedic Surgery, Sheba Medical Center, 2 Derech Sheba, Ramat Gan 52621, Israel; b Department of Orthopedic Surgery and Traumatology, Wirbelsäulenzentrum Ostschweiz, Brauerstrasse 95a, St. Gallen 9016, Switzerland; c Department of Research and Development, LEM Surgical AG, Morgenstrasse 136, Bern 3018, Switzerland; d Department of Neurosurgery, University Hospital Inselspital Bern, Rosenbühlgasse 25, Bern 3010, Switzerland

**Keywords:** Spine surgery, Intervertebral motion, Vertebral stabilization, Surgical robotics, Pedicle screw accuracy, Cervical spine, Biomechanics, Instrumentation/Navigation/Robotics

## Abstract

**Background:**

Navigation and robotic systems in spine surgery rely on the assumption that the spine behaves as a rigid body during surgery; however, intervertebral motion persists under typical intraoperative conditions. For robotic execution of hard-tissue procedures to be effective, the system must be capable of autonomously securing and stabilizing the target vertebra throughout the intervention.

**Methods:**

A cadaveric study was performed using 6 human cervical spine specimens. In part 1, C2–C7 vertebrae were instrumented with markers and subjected to robotically applied 30 N forces along planned pedicle screw trajectories, with and without robotic stabilization. Intervertebral motion was quantified using a 3D optical tracking system. In part 2, surgeons placed 30 cervical pedicle screws (C1–T1) using standard robot-assisted navigation and 36 screws using a novel FDA-cleared bi-manual 2-armed surgical robotic system incorporating autonomous vertebral stabilization and individual vertebra tracking. Screw accuracy was assessed by comparing planned and actual trajectories on postoperative 3D imaging.

**Results:**

Robotic stabilization reduced translational motion by up to 57.8% and rotational motion by up to 57.4% at the targeted level, with meaningful reductions at adjacent levels and diminishing effects with increasing distance. No major pedicle screw placement errors (>2 mm or >2°) were observed with stabilization. Mean angular error decreased by 31.7% (1.5 ± 1.2° vs. 1.0 ± 0.5°).

**Conclusions:**

Bi-manual robotic stabilization significantly reduced intervertebral motion and improved pedicle screw placement accuracy. Targeted intraoperative stabilization may mitigate limitations associated with spinal nonrigidity in robotic-assisted navigation.

## Introduction

Navigation and robotics in spine surgery use advanced imaging technologies, like CT and fluoroscopy, to improve surgical precision and accuracy. Among these, 3D imaging has been shown to outperform 2D and freehand techniques [[1], [2], [3]], providing superior accuracy in implant placement and navigation. In the lumbar spine, navigated pedicle screw placement yields significantly higher accuracy and safety than freehand techniques [4,5]. Despite these advancements, the widespread adoption of navigation and robotics in spine surgery remains limited due to several factors.

A major limitation is the inherent assumption in navigation and robotic systems that the spine behaves as a rigid body during surgery [6]. In reality, small intervertebral movements caused by respiration [7], surgical instrumentation, patient positioning, and underlying instability are inevitable [8,9]. This nonrigid behavior is a key source of inaccuracy because the virtual anatomical model in the navigation system no longer matches the true anatomy. These movements cause the spine to shift after registration, leading to registration errors and subsequent implant misplacement, and, when high-speed tools are navigated for bone removal, increase the risk of patient harm.

Consequently, the problem of intervertebral movements and the resulting inaccuracy of navigation persists. In the highly mobile cervical spine, emerging evidence indicates that navigated pedicle screw placement does not clearly outperform the freehand technique. A systematic review and meta-analysis by Bindels et al. [10] found no superiority of navigation over freehand placement in 4,697 cervical pedicle screws. Similarly, Irmak et al. [11] reported only a minimal difference in accuracy between freehand (85%) and navigation (83%) across 14,000 cervical pedicle screws. Taken together, these findings suggest that in the cervical spine, improved accuracy cannot be assumed from navigation alone. For a surgical robot to be effective in hard tissue procedures, it must therefore be capable of autonomously securing and stabilizing the bone prior to robotic intervention.

Our current study introduces a novel stabilization method utilizing a surgical humanoid bimanual robotic system that received FDA clearance. The architecture comprises an upper-torso humanoid platform equipped with 2 primary operating robotic arms and a tertiary arm dedicated to navigation and tracking. These 3 components operate in unison to provide a new cohesive stabilized environment: one robotic arm stabilizes a target vertebra while another performs the surgical tasks. The objectives of this study were to (1) investigate relative motion between cervical vertebrae when a force is applied to each vertebra as would occur during pedicle screw placement, (2) quantify the impact of intervertebral movement on navigated surgical accuracy, and (3) demonstrate how this novel robotic vertebral stabilization technique can improve accuracy by reducing spinal segment motion. We focused on assessing feasibility and preliminary efficacy of this stabilization method in reducing intervertebral movement in the cervical spine (C2–C7), the most mobile part of the spine.

## Methods

**Fig. 1:**
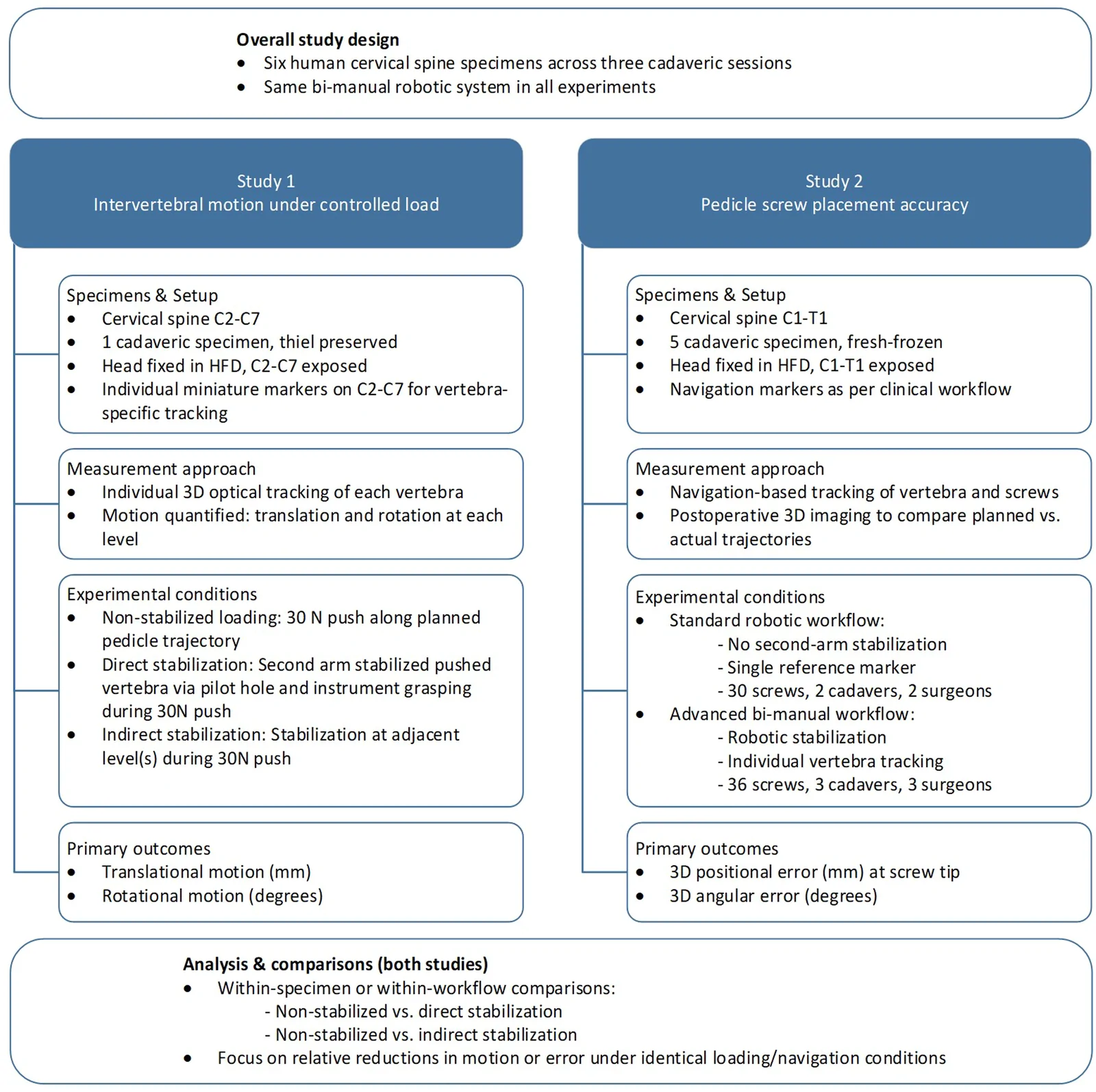
Schematic workflow of cadaver experiments and stabilization paradigms.

This cadaveric laboratory study was conducted across 3 experimental sessions using 6 adult human cervical spine specimens and comprised 2 complementary experimental components designed to evaluate the same stabilization concept from mechanistic and workflow perspectives. The first component quantified intervertebral motion under controlled loading with and without robotic stabilization, and the second evaluated the effect of the stabilization workflow on navigated cervical pedicle screw placement accuracy. To clarify how the different cadaver sessions, preservation techniques, tracking strategies, stabilization paradigms, and surgeon cohorts relate to each component, the experimental design is summarized in a schematic workflow (Fig. 1). Each component was conducted and analyzed according to its predefined objective, setup, and outcome measures, while both used the same bimanual robotic platform and stabilization principle.

Six adult human cervical spine specimens were used. Study 1 was performed in a single male specimen; no further demographic data were available. During the experiment it was noted that C4–C5 were naturally fused. In Study 2, the standard workflow cohort included 2 specimens (1 male, age 77 years; 1 female, age 77 years), and the advanced workflow cohort included 3 specimens (2 male, ages 92 and 72 years; 1 female, age 79 years). Bone mineral density was not measured, and no osteoporosis or prior cervical pathology were documented in any specimen.

All cadavers were positioned prone with the head secured in a head fixation device (HFD). The spinal area of interest was fully exposed without resection of the interspinous and the intertransverse ligaments, and 1 or more navigation markers were attached to the spinous processes of vertebrae. Intraoperative 3D scanners (CBCT, GE Healthcare Technologies Inc., Chicago, Illinois and Siemens AG, München, Germany) were used to register the positions of the navigation markers relative to the vertebrae. Clinically relevant pedicle screw trajectories were planned preoperatively in all scanned vertebrae using proprietary software of the robotic system (Dynamis Robotic Surgical System, LEM Surgical AG, Bern, Switzerland), enabling repeatable robot positioning relative to each vertebra. The bimanual robotic system comprises 2 primary robotic arms and a third component dedicated to navigation and tracking. In all experiments, 1 arm functioned as the working arm and the second served as the stabilizing arm. The stabilization concept was based on temporary rigid fixation of a selected vertebra through a pilot hole and instrument engagement, allowing 1 robotic arm to stabilize the target segment while the other applied force or performed screw-placement steps.

### Study 1: Individual spinal segment tracking during stabilized and nonstabilized force-loading-induced motion in C2–C7

The experiment was designed as a noncontrolled comparative study to characterize motion at levels C2 through C7 under externally applied loads, with and without robotic vertebral stabilization. Individual miniature markers were affixed to each vertebra from C2 to C7 using spinous-process clamps, and the 2 robotic arms were positioned bilaterally along preplanned cervical pedicle screw trajectories. Motion was recorded using a 3-dimensional optical tracking system with a stated positional accuracy of 0.1 mm (µCam RT optical tracking system, Axios 3D Services GmbH, Oldenburg, Germany). Use of individual vertebra markers enabled level-specific measurement of true vertebral motion rather than inferred movement from a single reference marker.

**Fig. 2:**
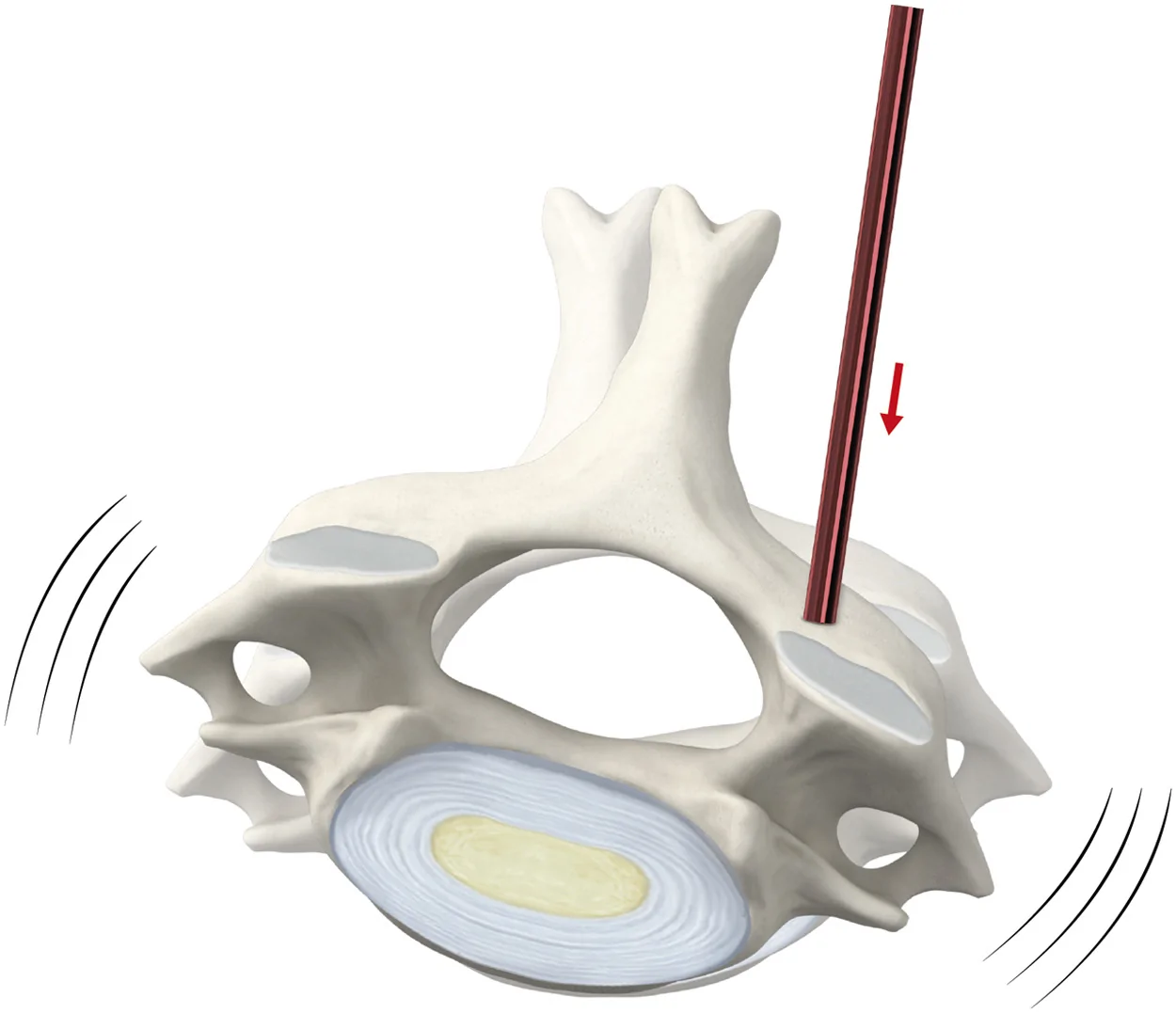
Illustration of the hypothetical effect of the 30 N push without stabilization. Red: robotically guided 30 N push force, resulting in vertebral movement.

The experiment was structured in 3 consecutive steps:

1. One robotic arm guided a uniaxial push force directed toward each cervical vertebra (Fig. 2, Fig. 3A). A target load of 30 N (±3 N) was selected based on preliminary measurements in a synthetic spine model simulating pedicle screw placement. A force gauge equipped with a long cylindrical rod was mounted to the robotic end effector, allowing translation along the defined trajectory while restricting off-axis movement. The controlled force was applied sequentially to each vertebra from C2 to C7, with 3 repeated pushes per level.

**Fig. 3:**
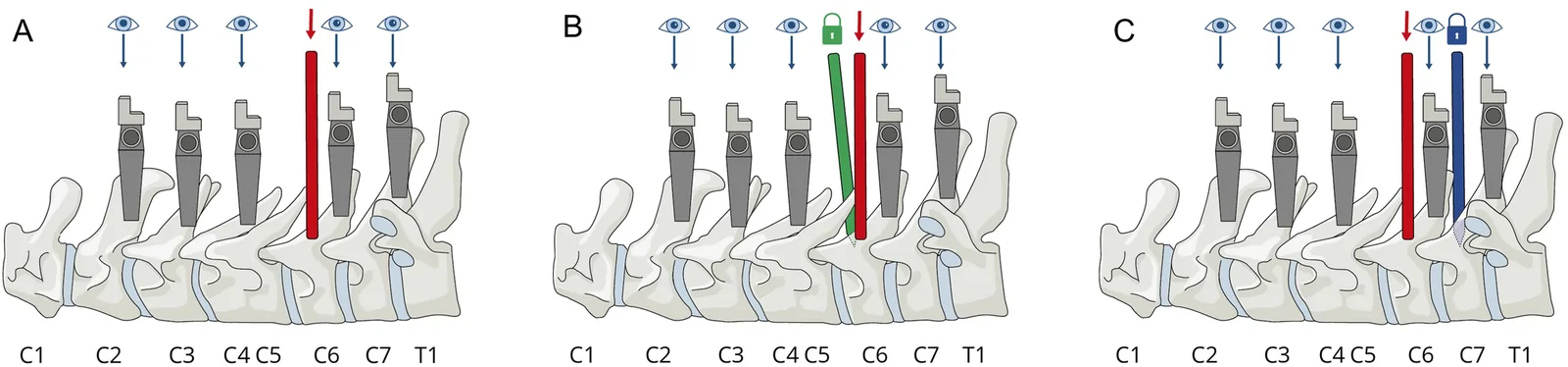
Graphical representation of cervical vertebrae with individually tracked navigation markers on C2–C7, observed on every level (eyes). Note that C4 and C5 are congenitally fused. (A) Uniaxial push force (30 N) is applied to the C6 level by the red cylindrical rod, no stabilization in place. (B) Vertebra C6 is directly stabilized by a second arm, grasping a tap that was placed in the pedicle illustrated by the green rod. (C) C6 is indirectly stabilized as illustrated by the blue rod at the vertebra 1-level away.
2. In the second part of the experiment, both robotic arms were used simultaneously: 1 arm guided a 30 N push force, while the other stabilized a selected vertebra during force application (Figs. 3B and C). For stabilization, a 3-mm pilot hole was first drilled along the planned pedicle trajectory through the robotic end effector. Subsequently, a spinal surgical instrument was inserted into the pedicle and rigidly grasped by the end effector. The preoperative screw trajectory planning and the magnitude and direction of the push force were not altered, ensuring that stabilized and nonstabilized cases were tested under identical loading conditions.
3. With this fixation in place, the same sequence of guided forces was reapplied from C2 to C7 while the marker displacements were recorded for comparison with the nonstabilized baseline. Fig. 4 illustrates the force-loading vector and stabilization location.

**Fig. 4:**
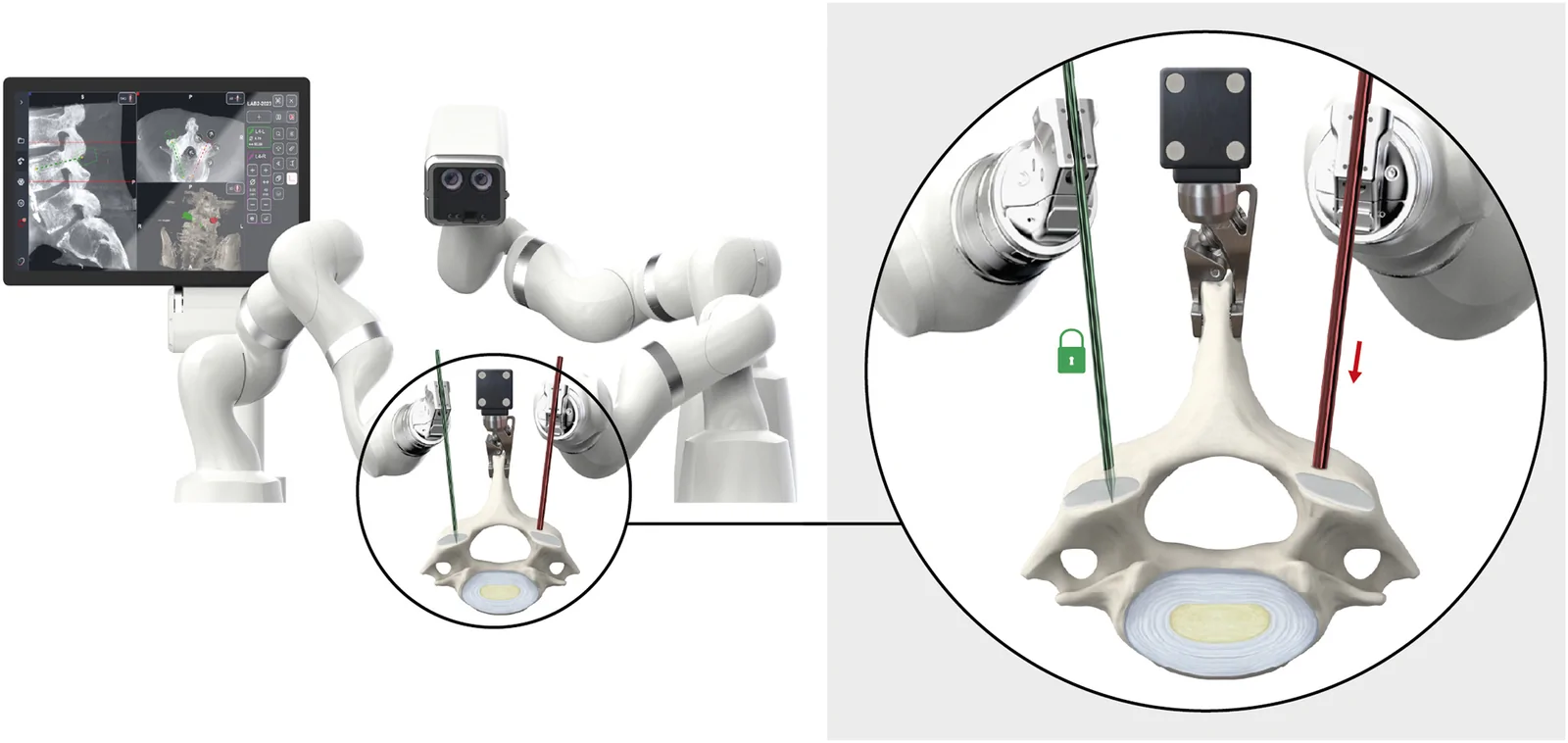
Visual representation of C7 stabilized by robotic arm (green), while another arm guides a 30 N push force (red). Contrary to Fig. 2, no movement is indicated due to the present stabilization.
4. Stabilized data was additionally recorded with the stabilization sequentially applied at C2–C7. For each condition, vertebral translation was quantified as the displacement between the marker’s start and end position across the 3 repeated pushes per vertebra. This is considered the primary outcome of the first study.

#### Data analysis of the individual spinal segment tracking

The raw data for a given tracked spatial rigid body M at time t are expressed as a translational vector $v_{t}\left(M,t\right)\;$ and a rotational quaternion q(M,t). The transformations measured were of the markers themselves, having their origin of the coordinate system 70 mm below the marker. This origin was considered clinically relevant, as it approximately corresponds to the tip location of a pedicle screw.

The quaternion q(M,t) is first converted into the equivalent rotation matrix $M_{q}\left(M,t\right)$, from which the rotation vector $v_{q}\left(M,t\right)$ and its Euclidian norm $∥v_{q}\left(M,t\right)∥$ are computed. The rotational motion of point M at time t is then defined as $∥v_{q}\left(M,t\right)∥$. For a given experiment, namely, 1 vertebra being stabilized while a vertebra is pushed, the rotational signal corresponds to the temporal evolution $∥v_{q}\left(M,t\right)∥\;=f_{q}\left(M,t\right)$. Similarly, the translational signal is defined as the temporal evolution $∥v_{t}\left(M,t\right)∥\;=f_{t}\left(M,t\right)$.

The signals are first smoothed using a Gaussian filter to facilitate the identification of time points at which the motion reaches its maximum; these points are referred to as peaks. Once the peak instants $t_{i}$ are identified, the corresponding peak values are extracted from the original signal, to avoid bias introduced by the smoothing step. The normalized amplitudes are averaged to obtain a single representative value. Peak detection parameters were manually tuned and the detected peaks were visually verified. All processing was performed in Python using automated scripts, including peak detection with the ‘find_peaks’ function from the SciPy library.

### Study 2: Pedicle screw placement accuracy in robotically stabilized and nonstabilized procedures

In the second study, the clinical workflow relevance of the stabilization concept was assessed by comparing cervical pedicle screw placement accuracy between a standard robot-assisted navigation workflow and an advanced bimanual stabilization workflow from C1 to T1. In the standard workflow cohort, 2 surgeons implanted 30 screws in 2 cadavers using robotic assistance without additional second-arm stabilization. In the advanced workflow cohort, 3 different surgeons implanted 36 screws in 3 cadavers using the same robotic platform with individual-vertebra tracking and robotic stabilization.

**Fig. 5:**
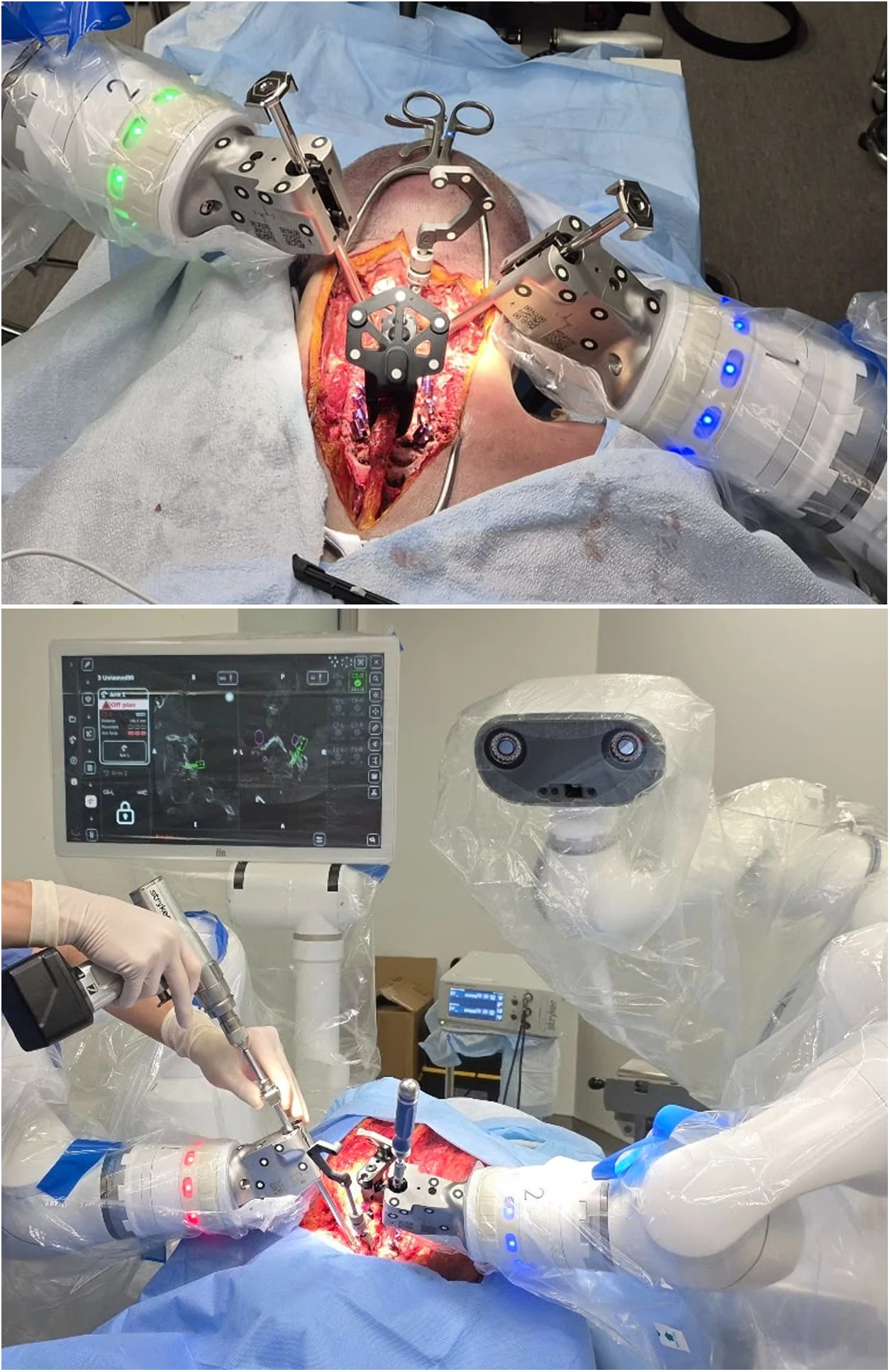
Photographs of stabilization workflow with the bi-manual robotic system. Top: blue LED indicates the stabilizing arm, stabilizing the vertebra with a drill cannula without tapping; green LED indicates the working arm, holding another drill cannula in place to drill the first pilot hole. Bottom: stabilizing arm stabilizes the vertebra with a tap inside the pedicle, while the surgeon manually drills through the other end effector.

The standard workflow reflected routine robot-assisted navigation without active stabilization by a second robotic arm. In contrast, the advanced workflow incorporated vertebra-specific tracking and bimanual stabilization during screw-path preparation and placement. Accuracy in both cohorts was assessed by comparing the planned screw trajectory from the preoperative 3-dimensional scan with the actual postoperative screw position using 3-dimensional image analysis software. Within the advanced workflow, direct and 1-level indirect stabilization techniques were alternated in a clinically relevant sequence. The first pilot hole on one side was drilled through one end effector while the second arm stabilized the vertebra by pressing with a drill cannula on the contralateral side. After tapping the pilot hole, the tap was rigidly grasped by the robotic arm. The contralateral pilot hole was then drilled, tapped, and grasped by the second end effector. The first tap was subsequently released and replaced by the definitive pedicle screw, after which the working sequence progressed to the next vertebra. Repetition of this pattern down the cervical spine generated an alternating sequence of direct and 1-level indirect stabilization. Fig. 5 shows the setup of the bimanual robotic system during the experiment.

The primary outcomes of this study were 3-dimensional positional error and 3-dimensional angular error between planned and actual screw trajectories. Positional error was measured at the screw tip, and angular error was defined by the difference between planned and actual screw centerlines on postoperative imaging. The frequency of screws exceeding 2 mm positional error or 2° angular error was also recorded as a clinically relevant threshold.

### Statistical analysis

Because this investigation combined multiple cadaver sessions and 2 complementary experimental components, the overall dataset was heterogeneous by design. Analyses were therefore performed within the experimental context in which the data were generated rather than as a single pooled analysis across all sessions. The first component used vertebra-specific optical tracking to characterize mechanical motion behavior under standardized loading conditions, whereas the second used a clinically oriented screw-placement workflow to assess accuracy outcomes. These different tracking strategies were intentional and aligned with the distinct objectives of the 2 components.

For the motion component, bilateral paired comparisons between stabilized and nonstabilized conditions were analyzed using 2-sided Student’s t-tests with 95% confidence intervals (CI) for the mean differences, fitting the small sample size. For the screw-accuracy component, differences between the standard and advanced workflows were evaluated using Mann–Whitney U tests, complemented by 95% bootstrap CI of the median difference. In addition, the distributions of the screw-accuracy outcomes were examined using Kolmogorov–Smirnov tests to assess deviations from normality and to support the choice of nonparametric methods. Given the exploratory cadaveric design and the modest sample sizes typical of navigation and robotics laboratory studies, the analyses were intended primarily to estimate the direction and magnitude of effects rather than to provide definitive clinical inference.

## Results

### Study 1: Individual spinal segment tracking during stabilized and nonstabilized force-loading-induced motion in C2–C7

**Fig. 6:**
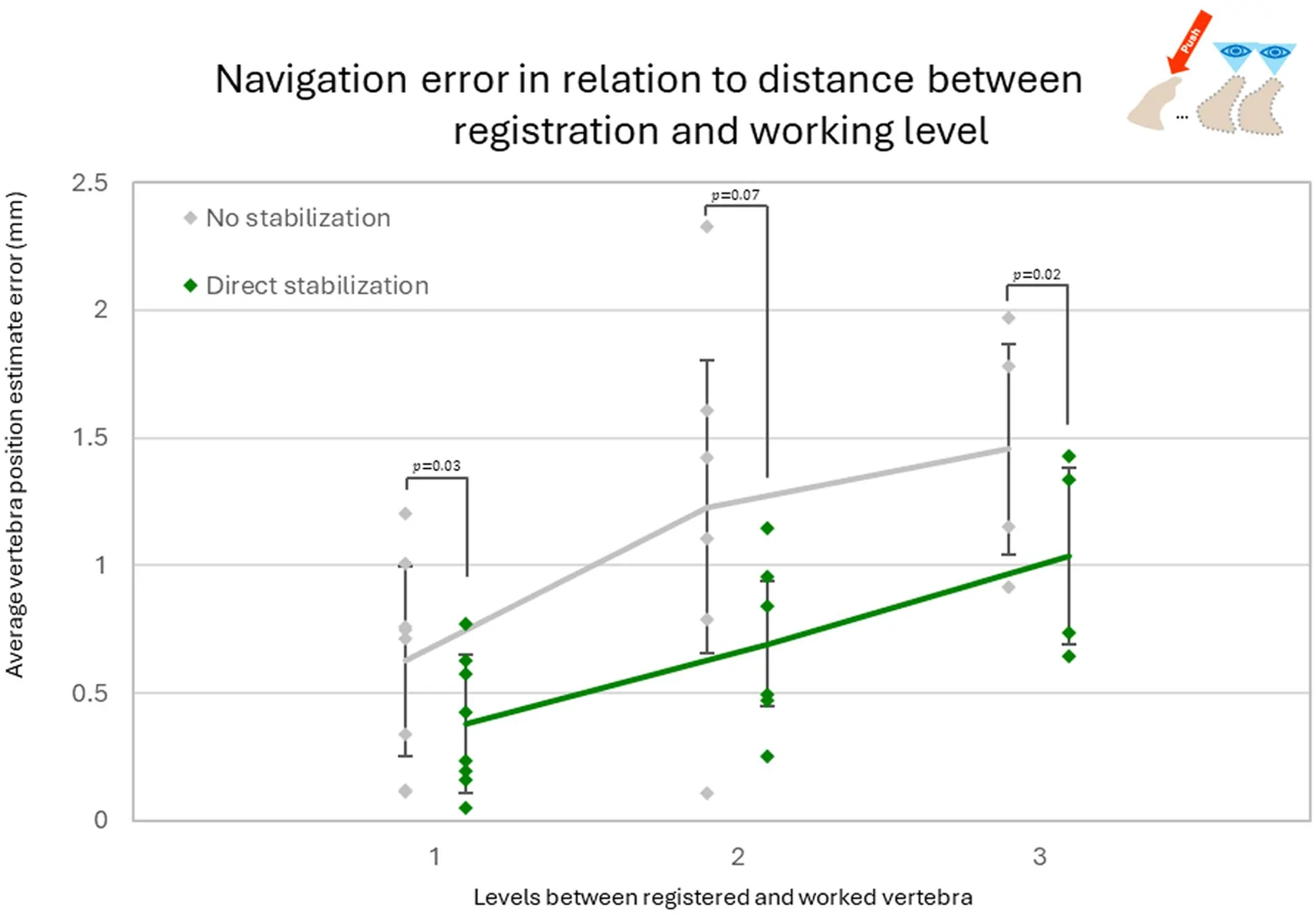
Mean navigation error in relation to the distance between the level of registration and the working level, normalized to the working level movement, including standard deviation. Stabilized vertebrae show lower errors than nonstabilized vertebrae, for both groups larger distances result in higher errors. The p-values are obtained from paired t-tests.

When motion of the force-loaded vertebra was compared with motion measured at vertebrae located 1 or more levels away, we found that greater intervertebral separation between the worked vertebra and the registered vertebra corresponded to larger errors between actual and registered movement. For example, nonstabilized force loading on C3 resulted in 2.8 mm translation at C3 (worked) and 2.0 mm at C2 (registered). Subtracting the movement, induced by the push force, which is visible for the surgeon in the navigation, the corresponding data point in the graph for the invisible movement is 0.8 mm at a 1-level distance. The results shown in Fig. 6 demonstrate that in a nonstabilized situation, average position estimate errors were 0.6 mm for 1 level distance, 1.2 mm at 2 level-distance and 1.5 mm at 3 level-distance, corresponding to 20%, 40%, and 60% of the actual induced motion, respectively. Direct stabilization reduced these errors to 0.38 mm (mean difference 0.25 mm; p = .03; 95% CI [0.04, 0.46]), 0.69 mm (mean difference 0.54 mm; p = .07; 95% CI [−0.07, 1.14]) and 1.04 mm (mean difference 0.42 mm; p = .02; 95% CI [0.11, 0.73]) for 1–3 level distances, respectively, with the 2-level comparison not reaching conventional statistical significance. Note that vertebrae C4 and C5 were congenitally fused and therefore treated as a single level.

**Fig. 7:**
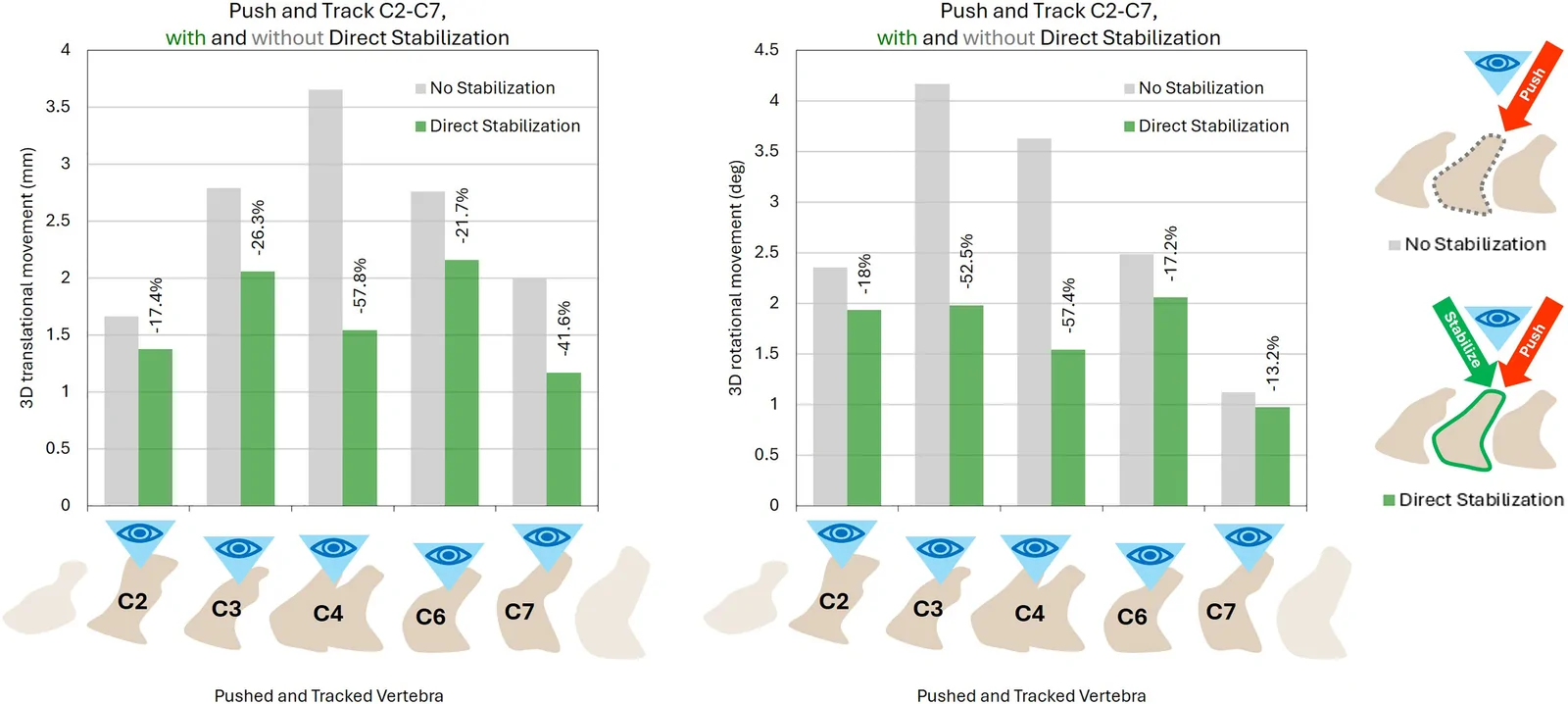
Relative motion, Translation and Rotation, for C2–C7 directly stabilized (green), plotted against nonstabilized vertebrae (grey). Percentages are calculated as the movement difference between the no-stabilization and stabilization conditions. Stabilized vertebrae moved 0.3–2.1 mm and 0.1–2.2° less than nonstabilized vertebrae. The graphic illustrates that C4–C5 were fused and therefore treated as a single level.

When measuring individual vertebral motion with and without stabilization using individual vertebra markers on C2-C7, we found for all levels, vertebral motion was lower in the stabilized condition than in the nonstabilized setup. Fig. 7 shows the translational and rotational movements of each vertebra in the stabilized and nonstabilized setups. Percentages represent the change in movement from the nonstabilized to the stabilized condition, negative values indicate reduced movement with stabilization, while positive values indicate increased movement. As mentioned before, C5 is absent from the horizontal axis due to a fusion between C4 and C5 in the specimen; therefore, these were treated as a single level (C4).

Direct stabilization of the force-loaded vertebra reduced translational movement by 0.3 mm at C2, 0.7 mm at C3, 2.1 mm at C4/C5, 0.6 mm at C6 and 0.8 mm at C7. Rotational movement decreased by 0.4° at C2, 2.2° at C3, 2.1° at C4/C5, 0.4° at C6 and 0.1° at C7 (absolute values in Fig. 7).

**Fig. 8:**
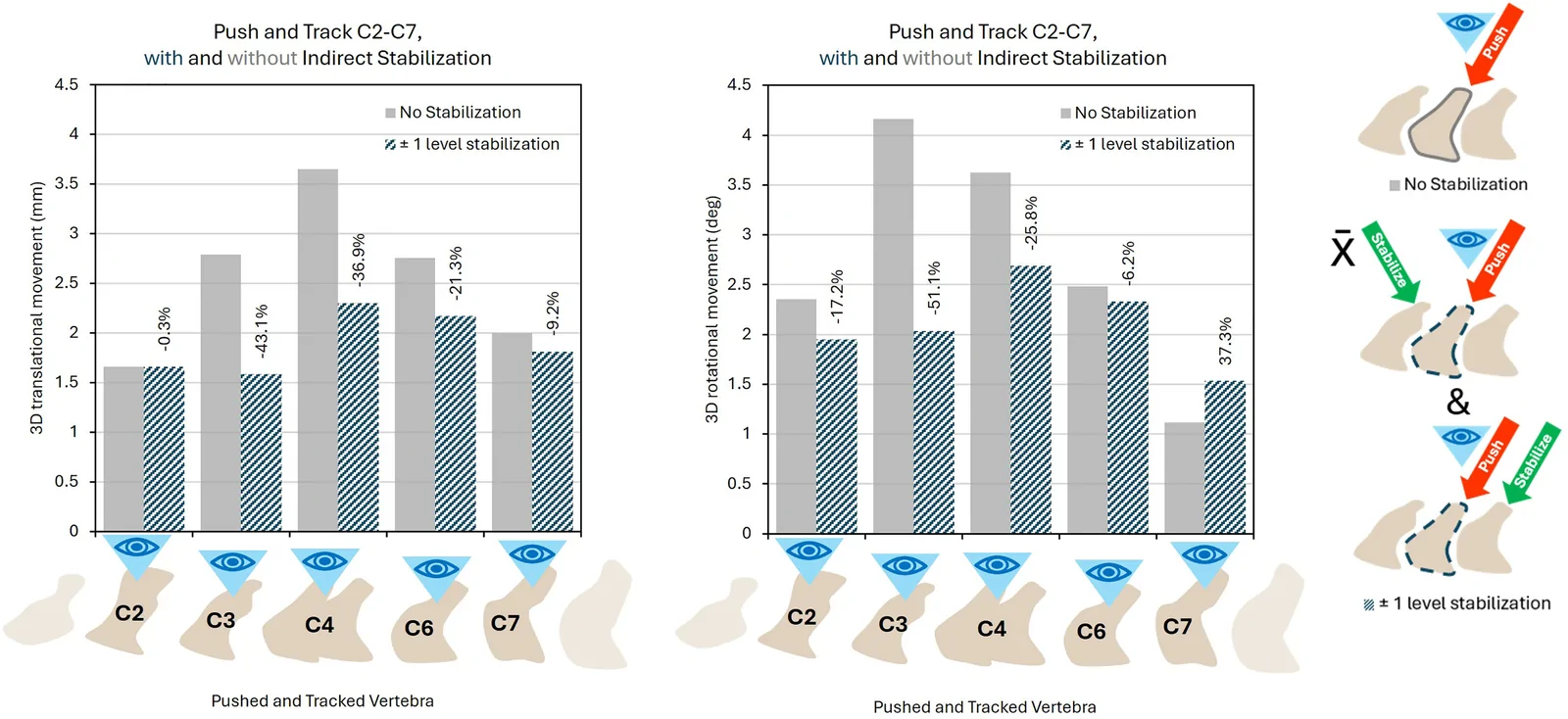
Relative motion, Translation and Rotation, for C2–C7 1-level indirectly stabilized (blue-dashed), plotted against nonstabilized vertebrae (grey). Percentages are calculated as the movement difference between the no-stabilization and stabilization conditions. Stabilized vertebrae moved 0.005–1.3 mm and 0.2–2.1° less, with 1 exception of 0.4° increase. The graphic illustrates that C4–C5 were fused and therefore treated as a single level.

Results for the 1-level indirect stabilization, in which the stabilized vertebra was 1 level away from the pushed vertebra, are shown in Fig. 8. The 1-level indirect stabilization reduced both rotational and translational motion in most cases. Reductions ranged from − 0.2° to − 2.1° for rotational movement and − 0.005 mm to − 1.3 mm for translation. There was only 1 exception where the 1-level indirect stabilization resulted in an increase of rotational motion, less than 0.4°. For C3–C6, 2 1-level indirect stabilization configurations were available (for example, C3 with stabilization on C4 and C2). Consequently, the movement value is an average of both. For C2 and C7 only one 1-level indirect stabilization configuration was implemented (with C3 and C6 stabilized, respectively). Also here, C5 is absent from the horizontal axis due to a fusion between C4 and C5 in the specimen; therefore, these were treated as a single level (C4).

**Fig. 9:**
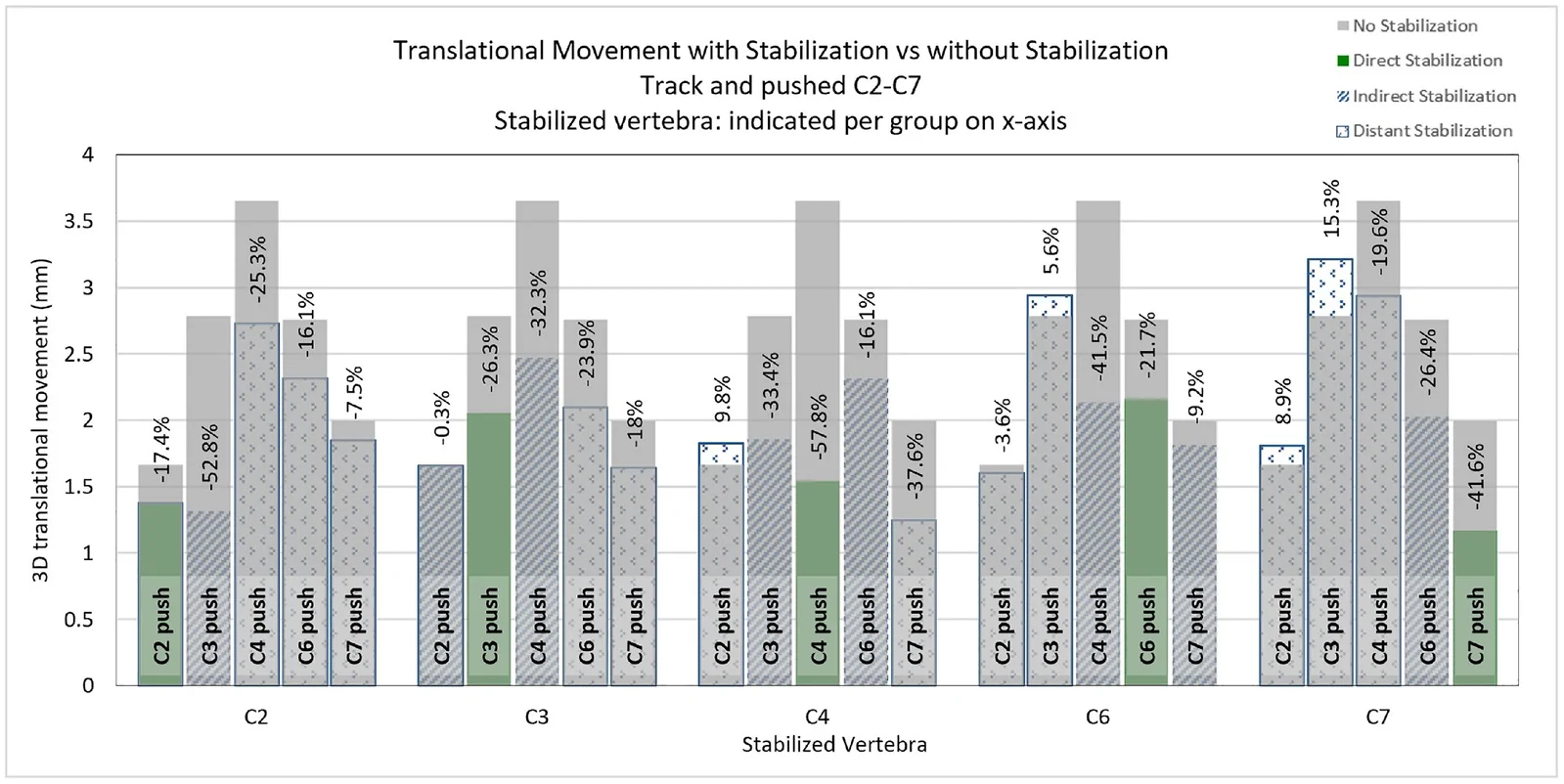
Rotational movement of vertebrae C2–C7 with different stabilization situations. Bars reflect actual motion of the pushed vertebra, individually tracked. Movement reductions of 0.15–2.19° have been found, with a few indirectly stabilized differences of 0.01–0.8° increase. C4 and C5 were fused and therefore treated as a single level.

**Fig. 10:**
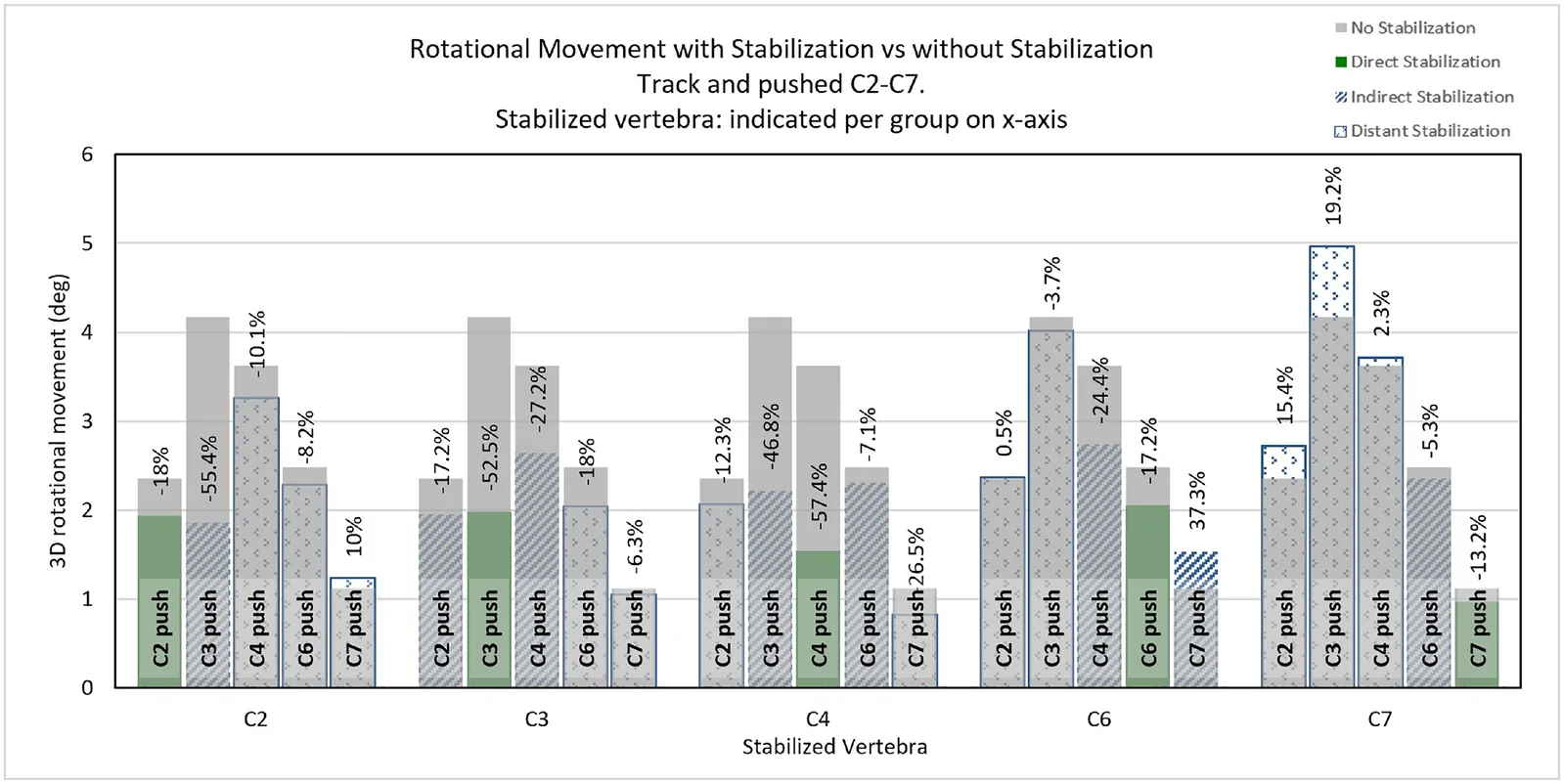
Translational movement of vertebrae C2–C7 with different stabilization situations. Bars reflect actual motion of the pushed vertebra, individually tracked. Movement reductions of 0.29–2.1 mm have been found, with a few indirectly stabilized differences of 0.14–0.43 mm increase. C4 and C5 were fused and therefore treated as a single level.

Fig. 9, Fig. 10 show the data of all tracked vertebrae. Overall, direct stabilization reduced both rotational and translational motion. Reductions ranged from 0.15° to 2.19° for rotation and from 0.29 mm to 2.1 mm for translation. For indirect stabilization (both 1-level indirect and distant stabilization), reductions ranged from 0.07° to 2.31° for rotational movement and from 0.005 mm to 1.18 mm for translation. However, a few indirectly stabilized cases showed greater motion than the nonstabilized condition, with differences of 0.01° to 0.8° in rotation and 0.14 mm to 0.43 mm in translation.

### Study 2: Pedicle screw position accuracy for robotically stabilized and nonstabilized procedures

**Fig. 11:**
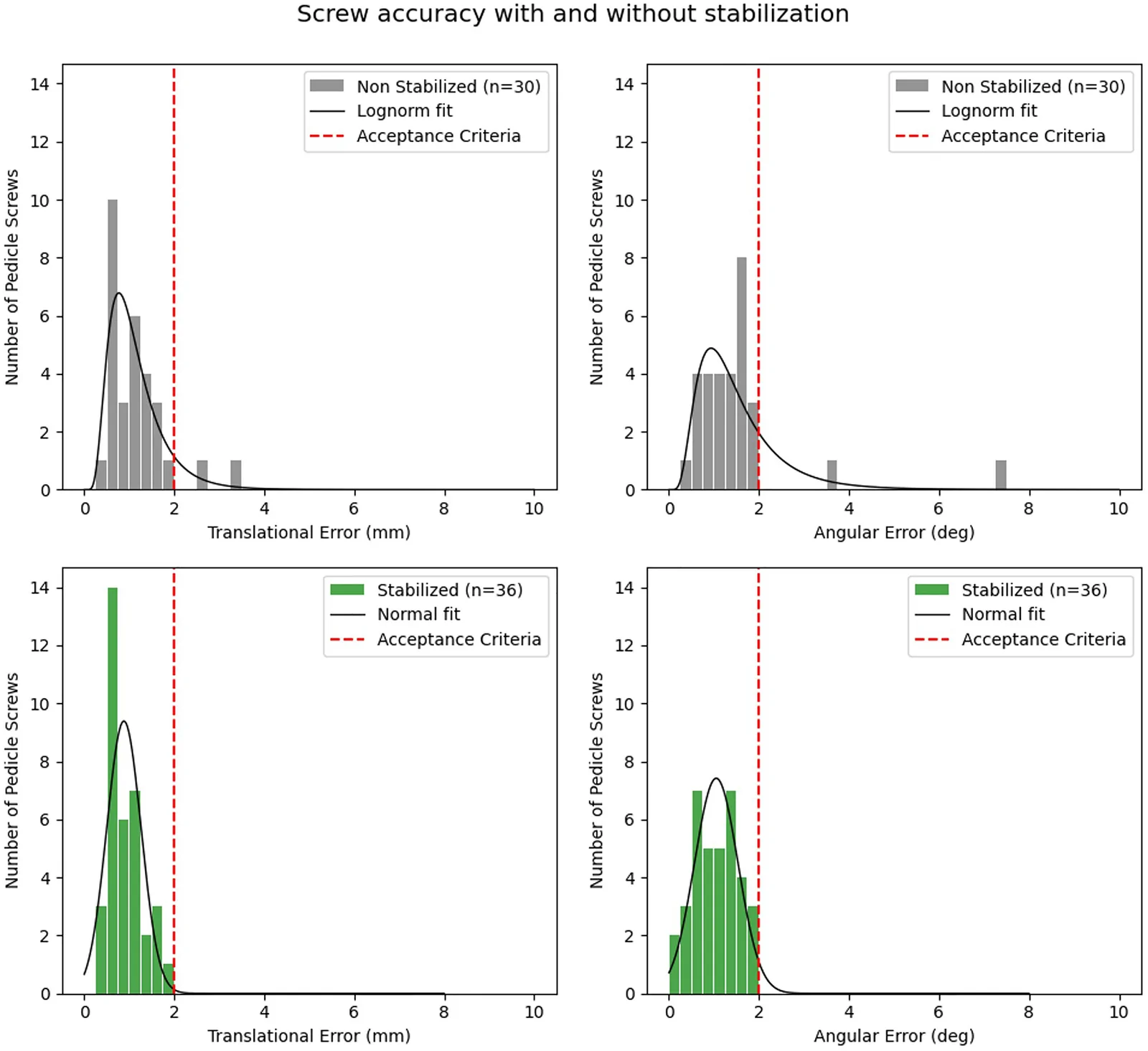
Histogram of pedicle screw accuracy, translation and rotation. Top: nonstabilized placement, bottom: stabilized placement. Red line: acceptance criteria for FDA clearance of robotic pedicle screw placement in the cervical spine. In the stabilized method, no critical errors above 2 mm and 2° occurred. The mean accuracy improved by 31.7% in rotation (p = .042), and 22.5% in translation (p = .099).

When observing the effect of the stabilized conditions in a clinically relevant surgical workflow performed by spine surgeons, a similar trend as above can be seen. Accuracy results are shown in Fig. 11: mean positional error decreased from 1.1 ± 0.9 mm to 0.9 ± 0.4 mm (22.5%), and mean angular error decreased from 1.5 ± 1.2° to 1.0 ± 0.5° (31.7%) with the novel stabilization workflow. The stabilized placement is compatible with a normal distribution (p-value >.01) while the nonstabilized placement is compatible with a lognorm distribution (p-value >.01). The Mann-Whitney tests show a significant median shift in the angular distribution (p = .04, 95% CI [0.02, 0.52]°), whereas the translational median difference obtained p = .10 (95% CI [−0.03, 0.43]mm), U statistics being equal to 698 and 668 respectively. Among the 36 stabilized screws, none had a positional error or angular error exceeding 2 mm or 2°. In the standard execution of 30 screws, 4 screws exceeded these thresholds.

## Discussion

This study evaluated cervical intervertebral motion under controlled loading and investigated whether intraoperative spinal stabilization could reduce vertebral displacement during robot-assisted procedures, thereby improving pedicle screw placement accuracy. The experiments showed that the robotic stabilization technique substantially decreased vertebral displacement, with the strongest effects at the directly stabilized level and at 1-level indirect stabilization, with diminishing effects at greater intervertebral distances. These findings support the premise that the cervical spine does not behave as a rigid body during surgery, and that external fixation can modify motion transmission patterns along the spinal column. The variability in the data underscores the differences in vertebral rigidity across levels. The presence of outliers, where rotation or translation increased with stabilization, indicates that additional investigation is needed to understand better what factors can affect stabilization at distant segments.

### The influence of biomechanics

The cervical spine is uniquely influenced by the morphology of the intervertebral joints. Bogduk and Mercer [12] describe these joints as saddle joints, a configuration that permits vertebral rotation around the transverse axis and additional rotation (sideto-side) around an axis perpendicular to the facets. This geometry provides the basis for traditional descriptions of cervical motion, whereby flexion–extension is generated through rotation about the transverse axis, and axial rotation and lateral flexion are inherently coupled due to the approximately 45° orientation of the facet-perpendicular axis relative to both conventional planes of motion [12]. The orientation of the facets differs between the upper and the lower cervical levels [12]. Quantitative kinematic data support this model: Mimura et al. [13] demonstrated that axial rotation in the lower cervical spine (C3–C7) averages 4–8° per segment and is consistently accompanied by comparable lateral bending. Their findings also highlight distinct kinematic behavior above the C2–C3 motion segment and confirm that the greatest proportion of cervical motion occurs at the craniovertebral junction, particularly between the occiput and C2 [13]. The diversity in facet orientation and the biomechanical coupling between the instrumented and stabilized vertebra could be one reason for the heterogeneous rotational and translational effects observed in indirect stabilized conditions. These relationships become more complex as the distance from the stabilized level increases, which may explain the variable motion patterns at distant segments.

### Navigation registration inaccuracy

Intervertebral motion alone can introduce clinically significant navigation errors when the spine is modeled as a rigid body. Intersegmental motion has been reported to result in pedicle screw deviations of approximately 3–6 mm, which are coherent with the translation magnitudes observed during this study and sufficient to cause severe pedicle wall breaches despite accurate initial registration [14]. Respiration [8], table motion, and reference frame displacement can rapidly degrade navigation accuracy. Even small postregistration movements have been associated with errors exceeding 2 millimeters and angular deviations of 3–5° [15]. These magnitudes are clinically relevant, especially in narrow cervical (4–5.5 mm, C3–C6 [16]) and thoracic (3.5–5 mm, T1–T8 [17]) pedicles [18].

Surgical manipulation further amplifies these errors. One study reported vertebral body motion exceeding 10 mm during maneuvers simulating soft-tissue dissection and pedicle targeting [8]. Another study found that intervertebral motion caused 18% severe pedicle breaches when the entire lumbar spine was registered as a single segment [14].

Even with intraoperative 3D imaging, deviations between planned and actual screw trajectories continue to occur due to post‑imaging motion and manipulation. Reported navigation errors following such disturbances commonly ranged from 2 to 6 mm, underscoring the fragility of rigid-body assumptions [18]. Guha et al. [9] demonstrated that navigation error in 3D spinal navigation propagates intraoperatively as a function of distance from the reference frame, intersegmental mobility, and respiration. Translational errors increased from submillimetric values near the reference to approximately 2–4 mm at distant levels, underscoring the dynamic degradation of navigation accuracy as the spine moves after registration [9]. The use of individual vertebra markers presented in this study is expected to compensate for these sources of error, suggesting that the remaining rotational and translational errors originate from other factors.

### Current stabilization techniques used for navigation or robot assisted screw placement

Robotic platforms often improve reproducibility of implant placements, but their accuracy still depends on maintaining alignment between the robot’s preoperative plan and intraoperative patient anatomy in the presence of intervertebral mobility. Several strategies have been proposed to mitigate navigation inaccuracies related to intervertebral motion. They include 3D-printed guide templates, registering each vertebra individually [19], static frames [20] or HFDs [21,22] with limited use and effect in the clinical setting because they are time-consuming [23] and lack real-time tracking capabilities [24]. The most common method to acquire tracking accuracy, in relation to anatomy, is the use of a rigid pin or clamp attached to the spinous process or iliac crest (for lumbar spine), which serves as the reference for navigation. This approach is standard across major robotic platforms and considered to be essential for accurate screw trajectory guidance [[25], [26], [27]]. However, it serves as a prerequisite for navigation rather than a sufficient sole measure to maintain accuracy throughout surgery and across the entire operated area.

Additional techniques include head fixation techniques such as positioning in the Mayfield or Doro-clamp for cervical spine procedures. Surgeons also use K-wires for temporary stabilization to “prestiffen” the construct before definitive screw placement. Bilateral anchored wires (left/right pedicles) can reduce micromotion of posterior elements relative to the operative trajectory during cannula exchanges and drilling, particularly in minimally invasive setups where soft tissue forces and instrument leverage can transmit motion. A correlation between K-wire placement accuracy and final screw accuracy has been reported in navigated cervical pedicle workflows [28].

Even with perfect registration, the act of creating a screw pathway may introduce error through skiving, elastic deformation, or motion of fracture fragments. Practical measures include sequencing instrumentation from levels closest to the reference frame outward and avoiding destabilizing maneuvers (eg, releases or osteotomies) before screw placement when possible [29]. It also includes using a high-speed drill to minimize applied force and thus dislocation/fracture fragment motion relative to the registered scan in percutaneous cervical fixation workflows.

Since accuracy can decay over time as anatomy shifts, many workflows rely on repeated intraoperative CT or cone-beam CT acquisitions (O-arm/iCT/CBCT) to verify and, if needed, repeat registration and “scan–place–confirm” loops, especially in in high-risk anatomy [30]. Large clinical navigation series using iCT-based navigation emphasize high accuracy but still note deterioration with increasing distance from the reference frame, implicitly supporting the need for reimaging along long constructs [30]. In the lumbar spine, single-level registration yielded 72% good, 24% fair and 4% poor for screw placements, whereas separate registration for each instrumented level improved results to 90% good, 10% fair and 0% poor screws [23]. A significant correlation has been demonstrated between pedicle screws misplacement and the distance from the registered level, particularly in the cervical spine. In a recent study, 6% of cervical screws were misplaced at the level of registration versus 50% at a 3-level distance, with range of motion (ROM) increasing up to a mean of 14.5° at 3 levels away [31]. Morello et al. [32] reported the highest accuracy (98%) using level-specific 3D-printed guide templates compared to 85% with navigation and 80% with freehand techniques. The stabilization technique studied in this paper not only indirectly reduces the need for multiple CT scans, thereby lowering resource usage during the surgical procedure, but also aims to counterbalance these applied forces during screw insertion to improve accuracy.

### Efficacy of the novel bi-manual workflow with robotic vertebral stabilization

Summarizing the quantitative cervical study, direct stabilization produced an average 35.5% reduction in translational movement at the direct stabilized vertebrae and 25.9% reduction at 1-level indirectly stabilized vertebrae. For rotational movement, reductions were 38.3% and 23.3%, respectively. One 1-level indirect scenario (C7 with stabilization at C6) showed increased rotation of 0.42° compared with direct stabilization. A hypothesis is that constrained degrees of freedom at the stabilization point, preventing translation, shifted more rotation around the stabilization axis. Because translation measured at the screw tip location decreased, this altered rotational behavior was not considered a reason to halt further investigation.

At distances of 2 or more segments, the benefit of stabilization diminished or even worsened compared to the nonstabilized condition. These findings align with those of Guha et al. [9] and suggest a constrained, but not completely rigid, mechanical coupling between vertebrae during surgical manipulation. The observation is consistent with clinical literature [31] reporting increasing pedicle screw misplacement with greater distance from the navigation reference.

Use of individual vertebral markers allowed differentiation between true and tracked movement. When each vertebra was tracked independently, unregistered motion of up to 1.4 mm was revealed, particularly significant when the tracked segment was more than 1 level away from the reference. The present findings indicate that the commonly utilized conventional head fixation device [22,33] may not provide adequate stabilization of cervical vertebrae. This observation is consistent with the unique anatomical and biomechanical characteristics of the cervical spine, which exhibits its greatest range of motion at the craniovertebral junction, particularly between the occiput and C2 [13]. The inherent mobility of this region likely contributes to the limited ability of standard HFD configurations to achieve truly stable fixation. Additionally, the results confirm that stability inferred from a single global marker may underestimate true vertebral motion and highlight the need for multilevel tracking and dynamic compensation algorithms in robotic systems.

Most importantly, translation of this stabilization concept to a simulated clinical workflow demonstrated meaningful improvements in surgical accuracy. Pedicle screws inserted using the advanced workflow (stabilization plus individual vertebra tracking) showed a significant 36.7% reduction in mean angular error and substantial reductions in variance: no screw trespassed the threshold of 2 mm and 2°.

Although the advanced method consistently showed lower translational and angular errors than the standard method, the nonparametric test for translation difference (p = .10) did not reach the conventional 0.05 significance threshold. For angular error, the Mann–Whitney test supported a statistically and potentially clinically meaningful reduction with the stabilized workflow (p = .04). This higher level of control over spinal motion may, in turn, facilitate new robotic applications that demand particularly high accuracy and safety margins, including robot‑assisted decompression. Incorporation of stabilization systems may therefore help overcome a key limitation of current robotic spine surgery.

### Study limitations

This study has several limitations that should be considered when interpreting the findings. First, this was an exploratory cadaveric investigation performed across multiple laboratory sessions where factors such as specimen preservation technique, bone density variation, surgeon cohorts and minor differences in screw trajectory planning may have introduced variability in the observed motion and screw-accuracy outcomes. Second, the sample size was modest as is typical for cadaveric navigation and robotics studies, which limits statistical power and precludes strong conclusions regarding generalizability or clinical effectiveness. Third, multiple surgeons participated in the screw-placement study, and each surgeon performed only 1 workflow arm rather than both conditions. As a result, surgeon-specific factors, including familiarity with cervical pedicle screw placement, technical preferences, and workflow adaptation, could not be separated fully from the effect of the stabilization strategy itself.

In addition, the screw-accuracy component focused on cervical pedicle screws from C1 to T1, whereas lateral mass screws are commonly used in many subaxial cervical procedures. Accordingly, the findings should be interpreted as specific to a pedicle screw workflow and should not be assumed to apply directly to other fixation strategies. The direction of applied force was dependent on preplanned trajectories rather than biomechanically standardized vectors. The sample size was modest, as is typical for cadaveric navigation and robotics studies, potentially limiting statistical power. Still, relative comparisons within identical loading configurations remain valid, and the multidataset design provides converging evidence for the observed stabilization effect.

Future work should include multispecimen cohorts, standardized force vectors, other cervical fixation techniques and evaluation of different stabilization methods. A clinical trial would be required to confirm intraoperative efficacy and validate patient-specific considerations such as deformity, soft-tissue tension, or osteoporosis.

## Conclusion

This exploratory cadaveric study confirms that clinically relevant intervertebral motion persists during cervical instrumentation and tends to increase with greater distance from the navigation reference level. When unmitigated, up to 2.0 mm of motion may remain undetected 3 segments away, potentially contributing to reduced screw placement accuracy. Within the constraints of this experimental model, targeted robotic vertebral stabilization reduced vertebral motion under load with maximal benefit at the directly stabilized level. When integrated into a navigation-assisted surgical workflow with individual vertebra tracking, stabilization was associated with significant lower (31.7%) mean angular pedicle screw-placement error and diminished deviations beyond 2 mm or 2°. These findings support the concept that targeted intraoperative stabilization may help improve the reliability of navigation in the mobile cervical spine. Larger, controlled studies are needed to confirm these observations.

## Author contributions

**AF:** Conceptualization, Writing—review & editing, Supervision. **FK:** Formal analysis, Writing—review & editing, Resources. **SAO:** Conceptualization, Investigation, Methodology, Formal analysis, Writing—original draft, Methodology, Resources, Project administration. **YB:** Conceptualization, Investigation, Writing—original draft, Writing—review & editing, Funding acquisition, Resources, Project administration, Supervision. **XCM:** Conceptualization, Methodology, Investigation, Writing—original draft, Resources. **AR:** Formal analysis, Writing—review & editing, Methodology, Resources, Supervision.

## Ethical approval

This study was conducted using human cadaveric specimens. Ethical approval for the European study site was obtained from Cantonal Ethics Committee Bern, KEK-BE (Approval No. 2024-02273). According to institutional policy, the research performed in the U.S. did not require formal ethics committee approval.

## Funding

This work was supported by LEM Surgical AG.

## Declaration of competing interests

The authors declare the following financial interests/personal relationships which may be considered as potential competing interests: **AF:** Consulting: LEM Surgical AG, Advisory Board: LEM Surgical AG, Stock ownership: LEM Surgical AG. **FK:** Advisory Board: LEM Surgical AG, Stock ownership: LEM Surgical AG. **SAO:** Research support (salary, materials): LEM Surgical AG. **XCM:** Research support (salary, materials): LEM Surgical AG. **YB:** Research support (salary, materials): LEM Surgical AG, Patents: LEM Surgical AG, Board: LEM Surgical AG, Stock ownership: LEM Surgical AG. **AR:** Royalties: Fehling AG, Stock ownership: LEM Surgical AG, Byties AG.
